# 5-Chloro-2-(4-methyl­phen­yl)-3-methyl­sulfinyl-1-benzo­furan

**DOI:** 10.1107/S1600536813018400

**Published:** 2013-07-06

**Authors:** Hong Dae Choi, Pil Ja Seo, Uk Lee

**Affiliations:** a Department of Chemistry, Dongeui University, San 24 Kaya-dong, Busanjin-gu, Busan 614-714, Republic of Korea; b Department of Chemistry, Pukyong National University, 599-1 Daeyeon 3-dong, Nam-gu, Busan 608-737, Republic of Korea

## Abstract

In the title compound, C_16_H_13_ClO_2_S, the dihedral angle between the mean plane [r.m.s. deviation = 0.004 (2) Å] of the benzo­furan ring system and the 4-methyl­phenyl ring is 29.25 (8)°. In the crystal, inversion dimers linked by pairs of weak C—H⋯O interactions generate *R*_2_^2^(14) loops.

## Related literature

For the pharmacological activity of benzo­furan compounds, see: Aslam *et al.* (2009 ▶); Galal *et al.* (2009 ▶); Khan *et al.* (2005 ▶). For natural products with benzo­furan rings, see: Akgul & Anil (2003 ▶); Soekamto *et al.* (2003 ▶). For the crystal structures of related compounds, see: Choi *et al.* (2007 ▶, 2009 ▶).
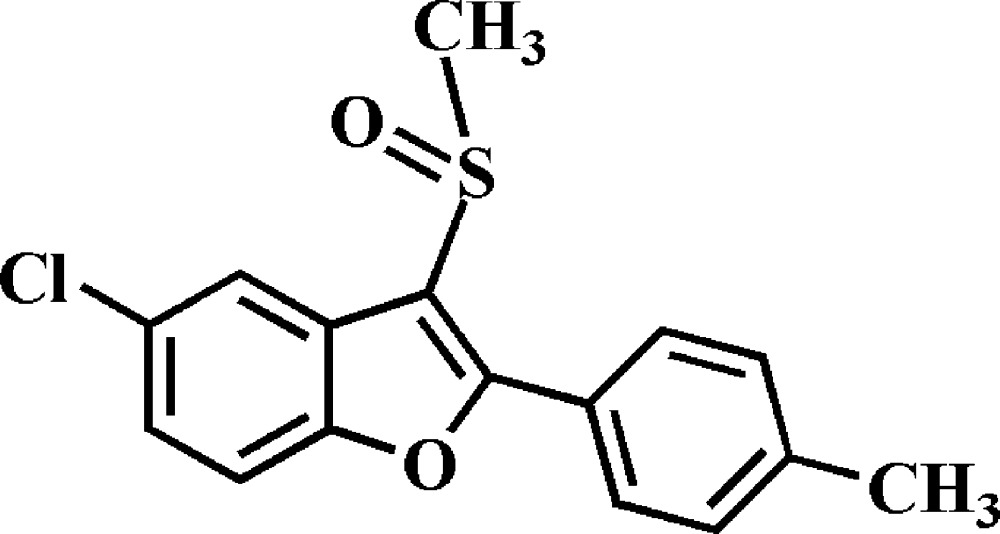


## Experimental

### 

#### Crystal data

C_16_H_13_ClO_2_S
*M* *_r_* = 304.77
Triclinic, 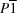

*a* = 8.0694 (8) Å
*b* = 8.0763 (8) Å
*c* = 11.4208 (11) Å
α = 90.185 (6)°
β = 96.280 (6)°
γ = 111.701 (6)°
*V* = 686.63 (12) Å^3^
*Z* = 2
Mo *K*α radiation
μ = 0.43 mm^−1^
*T* = 173 K
0.35 × 0.34 × 0.10 mm

#### Data collection

Bruker SMART APEXII CCD diffractometer
Absorption correction: multi-scan (*SADABS*; Bruker, 2009 ▶) *T*_min_ = 0.865, *T*_max_ = 0.958
12808 measured reflections
3423 independent reflections
3021 reflections with *I* > 2σ(*I*)
*R*_int_ = 0.048

#### Refinement

*R*[*F* ^2^ > 2σ(*F* ^2^)] = 0.038
*wR*(*F* ^2^) = 0.103
*S* = 1.05
3423 reflections
183 parameters
H-atom parameters constrained
Δρ_max_ = 0.32 e Å^−3^
Δρ_min_ = −0.28 e Å^−3^

### 

Data collection: *APEX2* (Bruker, 2009 ▶); cell refinement: *SAINT* (Bruker, 2009 ▶); data reduction: *SAINT*; program(s) used to solve structure: *SHELXS97* (Sheldrick, 2008 ▶); program(s) used to refine structure: *SHELXL97* (Sheldrick, 2008 ▶); molecular graphics: *ORTEP-3* for Windows (Farrugia, 2012 ▶) and *DIAMOND* (Brandenburg, 1998 ▶); software used to prepare material for publication: *SHELXL97*.

## Supplementary Material

Crystal structure: contains datablock(s) global, I. DOI: 10.1107/S1600536813018400/nk2211sup1.cif

Structure factors: contains datablock(s) I. DOI: 10.1107/S1600536813018400/nk2211Isup2.hkl

Supplementary material file. DOI: 10.1107/S1600536813018400/nk2211Isup3.cml

Additional supplementary materials:  crystallographic information; 3D view; checkCIF report

## Figures and Tables

**Table 1:** Hydrogen-bond geometry (Å, °)

*D*—H⋯*A*	*D*—H	H⋯*A*	*D*⋯*A*	*D*—H⋯*A*
C14—H14⋯O2^i^	0.95	2.54	3.436 (2)	158

Symmetry code: (i) 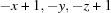.

**Fig. 1.**
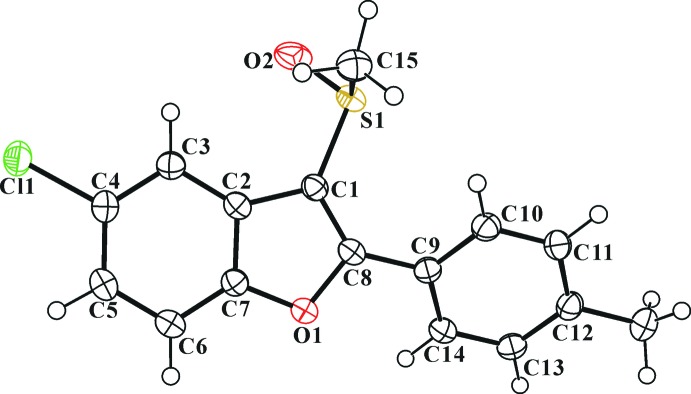
The molecular structure of the title compound. Displacement ellipsoids are drawn at the 50% probability level. H atoms are presented as small spheres of arbitrary radius.

## supplementary crystallographic information

### Comment

Many compounds having a benzofuran moiety have drawn much attention due to their valuable pharmacological properties such as antibacterial and antifungal, antitumor and antiviral, and antimicrobial activities (Aslam *et al.*, 2009, Galal *et al.*, 2009, Khan *et al.*, 2005). These benzofuran derivatives occur in a wide range of natural products (Akgul & Anil, 2003; Soekamto *et al.*, 2003).As a part of our continuing study of 5-chloro-3-methylsulfinyl-1-benzofuran derivatives containing phenyl (Choi *et al.*, 2007) and 4-fluorophenyl (Choi *et al.*, 2009) substituents in 2-position, we report herein the crystal structure of the title compound.

In the title molecule (Fig. 1), the benzofuran unit is essentially planar, with a mean deviation of 0.004 (2) Å from the least-squares plane defined by the nine constituent atoms. The dihedral angle between the mean plane of the benzofuran ring system and the 4-methylphenyl ring is 29.25 (8)°. In the crystal structure, molecules are connected by weak C—H···O hydrogen bonds (Table 1), resulting in a three-dimensional network.

### Experimental

3-Chloroperoxybenzoic acid (77%, 269 mg, 1.2 mmol) was added in small portions to a stirred solution of 5-chloro-2-(4-methylphenyl)-3-methylsulfanyl-1-benzofuran (317 mg, 1.1 mmol) in dichloromethane (40 mL) at 273 K. After being stirred at room temperature for 4h, the mixture was washed with saturated sodium bicarbonate solution and the organic layer was separated, dried over magnesium sulfate, filtered and concentrated at reduced pressure. The residue was purified by column chromatography (hexane–ethyl acetate, 1:1 v/v) to afford the title compound as a colorless solid [yield 70%, m.p. 459–460 K; *R*_f_ = 0.49 (hexane–ethyl acetate, 1:1 v/v)]. Single crystals suitable for X-ray diffraction were prepared by slow evaporation of a solution of the title compound in acetone at room temperature.

### Refinement

All H atoms were positioned geometrically and refined using a riding model, with C—H = 0.95 Å for aryl and 0.98Å for methyl H atoms. *U*_iso_(H) = 1.2*U*_eq_(C) for aryl and 1.5*U*_eq_(C) for methyl H atoms. The positions of methyl hydrogens were optimized rotationally.

### Crystal data

**Table:**

C_16_H_13_ClO_2_S	*Z* = 2
*M**_r_* = 304.77	*F*(000) = 316
Triclinic, *P*1	*D*_x_ = 1.474 Mg m^−^^3^
Hall symbol: -P 1	Melting point = 459–460 K
*a* = 8.0694 (8) Å	Mo *K*α radiation, λ = 0.71073 Å
*b* = 8.0763 (8) Å	Cell parameters from 4424 reflections
*c* = 11.4208 (11) Å	θ = 2.7–28.1°
α = 90.185 (6)°	µ = 0.43 mm^−^^1^
β = 96.280 (6)°	*T* = 173 K
γ = 111.701 (6)°	Block, colourless
*V* = 686.63 (12) Å^3^	0.35 × 0.34 × 0.10 mm

### Data collection

**Table:**

Bruker SMART APEXII CCD diffractometer	3423 independent reflections
Radiation source: rotating anode	3021 reflections with *I* > 2σ(*I*)
Graphite multilayer monochromator	*R*_int_ = 0.048
Detector resolution: 10.0 pixels mm^-1^	θ_max_ = 28.4°, θ_min_ = 1.8°
φ and ω scans	*h* = −10→10
Absorption correction: multi-scan (*SADABS*; Bruker, 2009)	*k* = −10→10
*T*_min_ = 0.865, *T*_max_ = 0.958	*l* = −15→15
12808 measured reflections	

### Refinement

**Table:**

Refinement on *F*^2^	Primary atom site location: structure-invariant direct methods
Least-squares matrix: full	Secondary atom site location: difference Fourier map
*R*[*F*^2^ > 2σ(*F*^2^)] = 0.038	Hydrogen site location: difference Fourier map
*wR*(*F*^2^) = 0.103	H-atom parameters constrained
*S* = 1.05	*w* = 1/[σ^2^(*F*_o_^2^) + (0.0432*P*)^2^ + 0.3137*P*] where *P* = (*F*_o_^2^ + 2*F*_c_^2^)/3
3423 reflections	(Δ/σ)_max_ < 0.001
183 parameters	Δρ_max_ = 0.32 e Å^−^^3^
0 restraints	Δρ_min_ = −0.28 e Å^−^^3^

### Special details

**Table:**

Geometry. All esds (except the esd in the dihedral angle between two l.s. planes) are estimated using the full covariance matrix. The cell esds are taken into account individually in the estimation of esds in distances, angles and torsion angles; correlations between esds in cell parameters are only used when they are defined by crystal symmetry. An approximate (isotropic) treatment of cell esds is used for estimating esds involving l.s. planes.
Refinement. Refinement of F^2^ against ALL reflections. The weighted R-factor wR and goodness of fit S are based on F^2^, conventional R-factors R are based on F, with F set to zero for negative F^2^. The threshold expression of F^2^ > 2sigma(F^2^) is used only for calculating R-factors(gt) etc. and is not relevant to the choice of reflections for refinement. R-factors based on F^2^ are statistically about twice as large as those based on F, and R- factors based on ALL data will be even larger.

### Fractional atomic coordinates and isotropic or equivalent isotropic displacement parameters (Å^2^)

**Table:**

	*x*	*y*	*z*	*U*_iso_*/*U*_eq_	
Cl1	0.21613 (6)	−0.06293 (6)	0.00434 (4)	0.03430 (13)	
S1	0.79728 (5)	0.32171 (5)	0.40498 (4)	0.02612 (12)	
O1	0.30621 (15)	0.15287 (15)	0.50057 (10)	0.0257 (2)	
O2	0.82257 (18)	0.19866 (17)	0.31756 (13)	0.0401 (3)	
C1	0.5655 (2)	0.2423 (2)	0.41897 (14)	0.0239 (3)	
C2	0.4219 (2)	0.1456 (2)	0.32835 (14)	0.0236 (3)	
C3	0.4092 (2)	0.0990 (2)	0.20888 (14)	0.0259 (3)	
H3	0.5122	0.1328	0.1679	0.031*	
C4	0.2396 (2)	0.0014 (2)	0.15325 (14)	0.0269 (3)	
C5	0.0852 (2)	−0.0505 (2)	0.21040 (15)	0.0291 (3)	
H5	−0.0284	−0.1179	0.1680	0.035*	
C6	0.0970 (2)	−0.0040 (2)	0.32891 (15)	0.0282 (3)	
H6	−0.0061	−0.0370	0.3698	0.034*	
C7	0.2666 (2)	0.0929 (2)	0.38407 (14)	0.0241 (3)	
C8	0.4897 (2)	0.2427 (2)	0.52001 (14)	0.0238 (3)	
C9	0.5607 (2)	0.3179 (2)	0.63960 (14)	0.0238 (3)	
C10	0.7141 (2)	0.4725 (2)	0.66158 (15)	0.0280 (3)	
H10	0.7738	0.5314	0.5976	0.034*	
C11	0.7804 (2)	0.5414 (2)	0.77602 (15)	0.0296 (4)	
H11	0.8862	0.6458	0.7898	0.036*	
C12	0.6930 (2)	0.4586 (2)	0.87084 (15)	0.0282 (3)	
C13	0.5390 (2)	0.3061 (2)	0.84809 (15)	0.0281 (3)	
H13	0.4780	0.2489	0.9121	0.034*	
C14	0.4720 (2)	0.2353 (2)	0.73466 (14)	0.0256 (3)	
H14	0.3661	0.1309	0.7213	0.031*	
C15	0.7646 (3)	0.5323 (3)	0.99514 (16)	0.0390 (4)	
H15A	0.6653	0.5005	1.0436	0.059*	
H15B	0.8228	0.6624	0.9952	0.059*	
H15C	0.8522	0.4818	1.0276	0.059*	
C16	0.8112 (3)	0.5192 (2)	0.32875 (16)	0.0318 (4)	
H16A	0.9311	0.5749	0.3036	0.048*	
H16B	0.7896	0.6031	0.3815	0.048*	
H16C	0.7206	0.4877	0.2595	0.048*	

### Atomic displacement parameters (Å^2^)

**Table:**

	*U*^11^	*U*^22^	*U*^33^	*U*^12^	*U*^13^	*U*^23^
Cl1	0.0365 (3)	0.0364 (2)	0.0259 (2)	0.01030 (19)	−0.00156 (17)	−0.00172 (16)
S1	0.0199 (2)	0.0253 (2)	0.0326 (2)	0.00772 (16)	0.00336 (15)	0.00359 (15)
O1	0.0207 (6)	0.0284 (6)	0.0265 (6)	0.0073 (5)	0.0031 (4)	−0.0001 (4)
O2	0.0316 (7)	0.0302 (6)	0.0614 (9)	0.0115 (6)	0.0178 (6)	−0.0034 (6)
C1	0.0214 (8)	0.0241 (7)	0.0262 (7)	0.0090 (6)	0.0017 (6)	0.0007 (6)
C2	0.0218 (8)	0.0215 (7)	0.0281 (8)	0.0091 (6)	0.0014 (6)	0.0021 (6)
C3	0.0257 (8)	0.0260 (7)	0.0269 (8)	0.0107 (6)	0.0035 (6)	0.0021 (6)
C4	0.0303 (9)	0.0243 (7)	0.0255 (7)	0.0106 (7)	−0.0008 (6)	0.0001 (6)
C5	0.0252 (8)	0.0272 (8)	0.0315 (8)	0.0076 (7)	−0.0026 (7)	0.0006 (6)
C6	0.0226 (8)	0.0287 (8)	0.0329 (8)	0.0089 (7)	0.0031 (6)	0.0024 (6)
C7	0.0238 (8)	0.0237 (7)	0.0256 (7)	0.0098 (6)	0.0021 (6)	0.0015 (6)
C8	0.0201 (8)	0.0218 (7)	0.0290 (8)	0.0073 (6)	0.0021 (6)	0.0021 (6)
C9	0.0241 (8)	0.0240 (7)	0.0258 (7)	0.0117 (6)	0.0034 (6)	0.0014 (6)
C10	0.0301 (9)	0.0248 (7)	0.0282 (8)	0.0081 (7)	0.0072 (7)	0.0031 (6)
C11	0.0297 (9)	0.0234 (7)	0.0329 (8)	0.0065 (7)	0.0046 (7)	−0.0028 (6)
C12	0.0311 (9)	0.0278 (8)	0.0275 (8)	0.0132 (7)	0.0030 (7)	−0.0024 (6)
C13	0.0298 (9)	0.0296 (8)	0.0270 (8)	0.0123 (7)	0.0075 (7)	0.0048 (6)
C14	0.0228 (8)	0.0236 (7)	0.0301 (8)	0.0081 (6)	0.0040 (6)	0.0027 (6)
C15	0.0417 (11)	0.0422 (10)	0.0295 (9)	0.0116 (9)	0.0035 (8)	−0.0084 (7)
C16	0.0342 (10)	0.0272 (8)	0.0355 (9)	0.0118 (7)	0.0087 (7)	0.0073 (7)

### Geometric parameters (Å, º)

**Table:**

Cl1—C4	1.7462 (16)	C9—C10	1.393 (2)
S1—O2	1.4879 (13)	C9—C14	1.399 (2)
S1—C1	1.7648 (17)	C10—C11	1.388 (2)
S1—C16	1.7925 (17)	C10—H10	0.9500
O1—C7	1.3768 (18)	C11—C12	1.393 (2)
O1—C8	1.3790 (19)	C11—H11	0.9500
C1—C8	1.364 (2)	C12—C13	1.387 (2)
C1—C2	1.445 (2)	C12—C15	1.505 (2)
C2—C7	1.393 (2)	C13—C14	1.382 (2)
C2—C3	1.397 (2)	C13—H13	0.9500
C3—C4	1.381 (2)	C14—H14	0.9500
C3—H3	0.9500	C15—H15A	0.9800
C4—C5	1.396 (2)	C15—H15B	0.9800
C5—C6	1.387 (2)	C15—H15C	0.9800
C5—H5	0.9500	C16—H16A	0.9800
C6—C7	1.378 (2)	C16—H16B	0.9800
C6—H6	0.9500	C16—H16C	0.9800
C8—C9	1.459 (2)		
			
O2—S1—C1	106.64 (7)	C10—C9—C8	121.45 (14)
O2—S1—C16	106.07 (8)	C14—C9—C8	119.67 (15)
C1—S1—C16	97.73 (8)	C11—C10—C9	120.65 (15)
C7—O1—C8	106.62 (12)	C11—C10—H10	119.7
C8—C1—C2	107.24 (14)	C9—C10—H10	119.7
C8—C1—S1	126.49 (13)	C10—C11—C12	120.54 (16)
C2—C1—S1	125.89 (12)	C10—C11—H11	119.7
C7—C2—C3	119.27 (15)	C12—C11—H11	119.7
C7—C2—C1	105.00 (14)	C13—C12—C11	118.46 (15)
C3—C2—C1	135.73 (15)	C13—C12—C15	120.74 (16)
C4—C3—C2	116.75 (15)	C11—C12—C15	120.80 (16)
C4—C3—H3	121.6	C14—C13—C12	121.65 (16)
C2—C3—H3	121.6	C14—C13—H13	119.2
C3—C4—C5	123.29 (15)	C12—C13—H13	119.2
C3—C4—Cl1	118.71 (13)	C13—C14—C9	119.81 (15)
C5—C4—Cl1	118.00 (13)	C13—C14—H14	120.1
C6—C5—C4	120.20 (15)	C9—C14—H14	120.1
C6—C5—H5	119.9	C12—C15—H15A	109.5
C4—C5—H5	119.9	C12—C15—H15B	109.5
C7—C6—C5	116.26 (15)	H15A—C15—H15B	109.5
C7—C6—H6	121.9	C12—C15—H15C	109.5
C5—C6—H6	121.9	H15A—C15—H15C	109.5
O1—C7—C6	125.14 (14)	H15B—C15—H15C	109.5
O1—C7—C2	110.63 (14)	S1—C16—H16A	109.5
C6—C7—C2	124.23 (15)	S1—C16—H16B	109.5
C1—C8—O1	110.51 (14)	H16A—C16—H16B	109.5
C1—C8—C9	133.98 (15)	S1—C16—H16C	109.5
O1—C8—C9	115.51 (13)	H16A—C16—H16C	109.5
C10—C9—C14	118.87 (15)	H16B—C16—H16C	109.5
			
O2—S1—C1—C8	142.96 (15)	C1—C2—C7—C6	−179.96 (15)
C16—S1—C1—C8	−107.64 (15)	C2—C1—C8—O1	−0.17 (17)
O2—S1—C1—C2	−29.09 (16)	S1—C1—C8—O1	−173.43 (11)
C16—S1—C1—C2	80.31 (15)	C2—C1—C8—C9	−179.34 (16)
C8—C1—C2—C7	−0.40 (17)	S1—C1—C8—C9	7.4 (3)
S1—C1—C2—C7	172.92 (12)	C7—O1—C8—C1	0.68 (17)
C8—C1—C2—C3	179.75 (17)	C7—O1—C8—C9	−179.99 (12)
S1—C1—C2—C3	−6.9 (3)	C1—C8—C9—C10	29.6 (3)
C7—C2—C3—C4	−0.1 (2)	O1—C8—C9—C10	−149.54 (15)
C1—C2—C3—C4	179.69 (16)	C1—C8—C9—C14	−151.36 (18)
C2—C3—C4—C5	0.1 (2)	O1—C8—C9—C14	29.5 (2)
C2—C3—C4—Cl1	−179.18 (11)	C14—C9—C10—C11	1.5 (2)
C3—C4—C5—C6	0.2 (3)	C8—C9—C10—C11	−179.49 (15)
Cl1—C4—C5—C6	179.45 (12)	C9—C10—C11—C12	−1.0 (3)
C4—C5—C6—C7	−0.4 (2)	C10—C11—C12—C13	0.2 (2)
C8—O1—C7—C6	179.86 (15)	C10—C11—C12—C15	179.82 (16)
C8—O1—C7—C2	−0.94 (16)	C11—C12—C13—C14	0.3 (2)
C5—C6—C7—O1	179.43 (14)	C15—C12—C13—C14	−179.38 (16)
C5—C6—C7—C2	0.3 (2)	C12—C13—C14—C9	0.2 (2)
C3—C2—C7—O1	−179.29 (13)	C10—C9—C14—C13	−1.0 (2)
C1—C2—C7—O1	0.83 (17)	C8—C9—C14—C13	179.91 (14)
C3—C2—C7—C6	−0.1 (2)		

### Hydrogen-bond geometry (Å, º)

**Table:**

*D*—H···*A*	*D*—H	H···*A*	*D*···*A*	*D*—H···*A*
C14—H14···O2^i^	0.95	2.54	3.436 (2)	158

Symmetry code: (i) −*x*+1, −*y*, −*z*+1.
